# Perception of Tunisian Public Health Practitioners on the Role of Primary Health Care during the COVID-19 Pandemic

**DOI:** 10.3390/ijerph191711118

**Published:** 2022-09-05

**Authors:** Sarra Melki, Donia Ben Hassine, Dhekra Chebil, Sarra Nouira, Youssef Zanina, Ahmed Ben Abdelaziz

**Affiliations:** 1 Information System Direction, University Hospital of Sahloul, Sousse 4054, Tunisia; 2 Sousse Faculty of Medicine, University of Sousse, Sousse 4002, Tunisia; 3 Research Laboratory LR19SP01, Sousse 4054, Tunisia; 4 Monastir Faculty of Medicine, University of Monastir, Monastir 5000, Tunisia; 5 Monastir Faculty of Pharmacy, University of Monastir, Monastir 5000, Tunisia

**Keywords:** Primary Health Care, epidemics, COVID-19, qualitative research, Tunisia

## Abstract

Context: Primary Health Care is the first level of healthcare delivery services. Its role in the management of epidemics has been documented especially during the SARS and Ebola epidemics, and more recently during the COVID-19 pandemic. Objective: To describe public health experts’ perceptions of the implication of Primary Health Care on managing the COVID-19 pandemic in Tunisia. Methods: This qualitative study was based on a structured interview covering five domains: 1. Preparedness, 2. Implication, 3. Health delivery, 4. Response and 5. Fight against COVID-19 in Primary Health Care in Tunisia. Convenient sampling was done to include public health practitioners and experts. Results: A total of 25 experts were included with a sex ratio that was equal to 0.92, including two international experts, and four that were working in the Ministry of Health. The majority of respondents affirmed that the Tunisian PHC was not prepared to fight against the COVID-19 pandemic. Concerning the response role of PHC against COVID-19, some experts stated that PHC played an important role in the early stages of the pandemic. Almost all included participants claimed that PHC was marginalized from the national strategy against COVID-19. In addition, all respondents affirmed that there had been a weakening effect of the delivery of the minimum healthcare package that was dispended by the PHC after the pandemic. However, they all expressed the ability of PHC to manage future epidemics. Conclusion: The Tunisian PHC system did not play an efficient role in the current COVID-19 pandemic. However future lessons should be deduced for further implications in potential upcoming epidemics.

## 1. Introduction

Primary Health Care (PHC) is one of the most important pillars of universal health care as it is the population’s first contact with the health system [1]. It was defined by the WHO and UNICEF as a holistic approach to health, aiming to ensure an equitable distribution of the highest possible level of healthcare services from health promotion and disease prevention to treatment, rehabilitation, and palliative care [2]. The three main functions of PHC, as defined by both the Alma Ata in 1978 [3] and declared by the Astana in 2018 [4], are to provide an equitable package of health, to ensure communityparticipation (in health planning, organization, and control), and to encourage inter-sectoral collaboration (such as with education and agriculture sectors). Even though the role of PHC in the management of epidemics was absent in the Alma Ata declaration and briefly mentioned in Astana declaration, there have been historical experiences and recognition of the paramount role of PHC in reducing the impact of epidemics within the community such as during the SARS (2003) and Ebola epidemics (2013) [5,6].

The current COVID-19 pandemic, officially declared by the WHO on the 11March 2020, had immediate effects on societies, economic life, and the organization of the healthcare systems worldwide as it urged both PHC and hospitals to respond to challenges in new health needs and requirements. PHC was managing a great share of COVID-19 related care, despite the international focus being on third-line hospitals, and intensive care units [7]. Some PHC systems played a crucial part in the fight against COVID-19 and were able to manage cases and relieve the burden of secondary and tertiary care [8,9]. These examples suggest that countries with PHC expertise could better manage the COVID-19 pandemic, locally. Tunisia has a public health system that is funded by taxation, and it mainly includes three healthcare levels. The first level is the PHC centers which were created following the Alma-Ata declaration, and currently, there arearound 2000 PHC structures all across the country [10]. PHC centers provide the essential healthcare package including vaccination, maternal care, and health promotion. The second level is represented by the regional hospitals which provide medical, surgical, and obstetric care. The last level contains university hospitals that are characterized by hyper-specialized care, educational, and research functions. Despite the great number of PHC structures and the largeamount of expertise within PHC, the role of PHC in the management of COVID-19 remains unclear [11]. Therefore, the aim of this study is to describe public health experts’ perceptions of the role that PHC played during the COVID-19 pandemic in Tunisia in terms of preparedness, response, and management.

## 2. Methods

**Figure 1:**
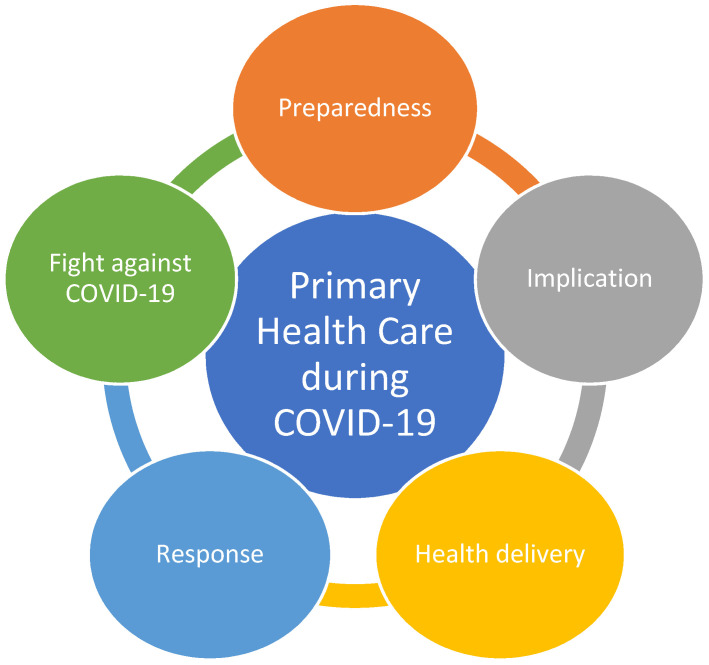
The five domains explored in the study describing the role of the Tunisian Primary Health Care in the management of the COVID-19 pandemic.

The study design was qualitative, using a structured interview covering the five domains illustrated in Figure 1.

The study population consisted of Tunisian Public Health Practitioners and experts ranging from juniors (Preventive and Community Medicine residents) to seniors (Preventive and Community Medicine professors, Ministry of Health workers, and international experts). Participants were invited to take part in the study by email and were selected using a convenient sampling method. All participants delivered their written consent to participate. A one-on-one interview was not possible due to COVID-19 protocols therefore, an online five-question structured questionnaire was sent via email to the participants (Box 1). Hypothetico-deductive and thematic analyses were used to analyze the collected data. The analysis of the qualitative data was done manually by two of the investigators. They went through the responses and selected the topics that were related to each of the five themes.

Alongside the qualitative data, some variables were collected such as sex (male, female, and others), age, and function (international: public health experts working in the locals of the WHO; national: public health practitioners working in the ministry of health; regional: working in regional directions of health; local: working in Primary Health Care centers or hospitals). The variable implication in PHC reflects the years of exercising as a public health practitioner (student: below 5 years; practicing: between 5 and 35 years; retired above 35 years).

Box 1 **The five-question questionnaire that was delivered to the participants of the study to describe the role of the Tunisian Primary Health Care in the management of the COVID-19 pandemic**

**Topic 1:**

In Tunisia, Primary Health Care (structures, programs, and staff) has been prepared for the fight against the COVID-19 pandemic during its different waves.

**Topic 2:**

In Tunisia, Primary Health Care (structures, programs, and staff) has played a significant role in the response to the COVID-19 pandemic and its various waves.

**Topic 3:**

In Tunisia, the national strategy to fight against the COVID-19 in its various waves has marginalized Primary Health Care in the provision of preparedness and response measures.

**Topic 4:**

In Tunisia, the national strategy to fight against COVID-19 has weakened the capacities of Primary Health Care in the usual dispensation of the minimum package of essential care.

**Topic 5:**

In the future, the fight against critical epidemics (such as COVID-19) is a component that can be integrated into the Primary Health Care strategy (essential health care, inter-sectoral collaboration, and community participation), in Tunisia.

## 3. Results

A total of 25 public health experts have responded to the questionnaire with a sex ratio (male to female) that was equal to 0.92 and a mean age that was equal to 51 ± 16 years. Twenty-three of the respondents were medical doctors, of which five were residents in Preventive and Community Medicine and two were WHO experts. The other characteristics are in Table 1.

Overall, the majority of respondents (23 participants) agreed that PHC in Tunisia was not prepared to fight against the COVID-19 pandemic. This is due to the fact that the national health system did not give enough importance to PHC, although the existence of a national strategy against epidemics has been elaborated since 2016. R H stated: “*A preparedness plan for response and resilience to diseases with epidemic potential in Tunisia, was developed in 2016, … However, to my knowledge, all the documents thus developed were not used during the COVID 19 pandemic …*”. The non-preparedness of PHC in Tunisia was manifested by a “*lack of protective equipments”* and a “*poor organization of resources”*.

Concerning the response role of PHC against the COVID-19 pandemic, the responses diverged into two main categories. On the one hand, some experts stated that PHC played an essential role in the fight against COVID-19 at an early stage. PHC was implicated in the “*epidemiological surveillance*”, “*diagnosis of COVID-19 cases*”, “*community information and sensitization*” and “*triage of patients*”. As for the vaccination against COVID-19, some experts mentioned that the role of PHC was very delayed due to the creation of dedicated vaccine centers. Dr H A mentioned that “*all of the occasional ad-hob vaccination centers were largely managed and operated by basic health care personnel, occasionally mobilized*”. On the otherhand, some experts claimed that PHC did not play a role in the fight against the COVID-19 pandemic as the delivery of care was centralized and some PHC structures were closed. “…*They chose excessive centralization and putting on hold of all activities (even the closure of the most peripheral centers) a way of marginalizing the first line and giving a signal of lack of confidence in these structures which have certainly played an important role in the 20th century*,…” was stated by T A.

The third domain of the questionnaire was concentrated on whether the national strategy against COVID 19 had marginalized PHC in the preparedness and response plans. Only one expert affirmed that “*the response strategy is adapted to epidemiological data and the WHO recommendations. The PHC was associated when the possibilities of response by hospitals and central structures were exceeded at the time of the summer wave of 2021 with the delta variant, which is particularly serious”,* Pr. A BH. Other experts claimed PHC was marginalized, and that the national decision-makers “*copied*” the strategy of some European countries, “*especially France*“.

Otherwise, all respondents affirmed that there has been a weakening in the delivery of the minimum healthcare package that is dispensed by PHC after the COVID-19 pandemic. This was due to the national political priority according to managing COVID-19 cases. “*It was a choice of the decision makers of the ministry of health and the presidency of the government, who considered that other diseases could wait … a COVID death was a failure”,* stated Pr M H, a professor in preventive and community medicine. Therefore, there has been an allocation of resources to manage the COVID 19 pandemic with “*staff reorganization*”, “*reservation of health structures for COVID 19 patients*” and making sure that there is a “*supply of oxygen and treatment for COVID 19 at the expense of antibiotics and drugs for chronic pathologies”.*

On whether or not the management of future epidemics should be a part of PHC in Tunisia, all included experts stated that they agree. Dr. S B, a WHO expert, affirmed that “*the strength of the Tunisian health system is its foundation on PHC despite the non-development of social and community participation and inter-sectoral coordination”.* However, several measures should be taken to improve the Tunisian PHC system such as the “*reinforcement of human resources*” and the “*development of the infrastructure*. Table 2 summarizes the responses and arguments of the 25 participants regarding the five themes of the questionnaire.

## 4. Discussion

The role of PHC in the management of previous epidemics has been described in the literature and it has highlighted the crucial role of PHC in the prevention, and treatment of non-severe cases. Despite the unpredictable occurrence of the current COVID-19 pandemic, the PHC system has shown its important role in itsprevention, and the screening and awareness of the community, and the management of positive cases.

This study is among the early first qualitative assessments exploring the role of PHC in the management of the COVID-19 pandemic in Tunisia. A standard qualitative study method, such as a one-on-one interview or a focus group, was not conducted due to the COVID-19 restrictions. However, the interview guide was distributed to the participants via emails which allowed them to respond freely with more temporal ordering, coherence, and self-reflection than oral accounts could. The selection of the participants was made via a convenient sampling method, which allowed including only public health practitioners whose electronic addresses were in the emailing list of the public health college. Nevertheless, our sample was heterogeneous and allowed us to include public health professionals ranging from students to WHO experts.

### 4.1. Non-Preparedness to Face the COVID-19 Pandemic

According to our results, the Tunisian PHC had an inefficient performance in the delivery of essential health care services and the management of the pandemic. The PHC system in Tunisia was established in the early 80s, following the Alma Ata declaration. However, twenty years after its launch, it “was exhausted, and even began to counter-produce”, said Professor Omar Brixi [12]. Long before the COVID-19 pandemic, the PHC system in Tunisia has been reduced to a set of preventive tasks such as vaccination, family planning, and school health as a result of the non-reactivation of the PHC by reforms, and the focus on hyper-specialization and hospital-orientation [12]. During the current pandemic, the PHC system encountered numerous challenges. First, its mission to deliver the essential health care package and the management of communicable and non-communicable diseases has been altered [13]. This may be due, to the community phobia that limited patients’ consultations, the absenteeism of healthcare workers, and the closure of some healthcare facilities [14]. The literature has also demonstrated that there have been changes in the demand and offer of PHC services characterized by a decrease in in-person visits, a drop-in vaccination rate, and a rise in non-communicable diseases’ complications [15,16,17]. Second, PHC in Tunisia, as highlighted by the participants of the study, did not play an effective role in the management of the COVID-19 pandemic. Even though the national political leaders declared COVID-19 as a health priority, the “while other diseases can wait” national strategy did not include the PHC system in the response plan, and it was focused on tertiary, hyper-specialized hospitals for the management of positive cases. This decision was partially influenced by the national scientific comity made by virologists, reanimations, and pulmonologists physicians (excluding public health experts) [18].

### 4.2. A Missed Opportunity

Our results have demonstrated that there has been an inequitable distribution of resources during the COVID-19 pandemic in favor of hyper-specialized centers and hospitals. In fact, the participants claimed that access to PHC was difficult during the pandemic, especially with the absenteeism of the staff and the closure of some centers as well as the limitation of treatments and remote appointments. For example, during the pandemic, the absenteeism of the staff was partially due to the lack of protective equipment in the PHC centers, and the decision to stop any type of non-urgent or routine care which led, gradually, to the closure of some PHC centers and the disruption the essential drugs’ availability. These challenges during the COVID-19 pandemic have tested the healthcare delivery systems around the globe and have exposed crucial deficiencies in the health system. Although there are still the existing challenges of the classic modes of healthcare delivery and access, new alternatives to healthcare delivery have emerged such as telemedicine [19,20]. Due to the lockdowns that were imposed by most countries and the community phobia and the drop in face-to-face consultations that followed, multiple changes in practice, management, and consultation strategies were quickly adapted, aswas characterized mainly by the switch toward telephone triage and consultations [21,22]. This method has partially contributed to the continuation of chronic care. Although the shift to telehealth has improved health access, issues with technology access and literacy arecommon. In Tunisia, telemedicine is regulated by the 2018-43 law [23]; however, the practice of telemedicine during the pandemic has been reduced to private practitioners. PHC did not shift its practice to a more innovative method, and this may be due to poor infrastructure and lack of technological supplies. For the vaccination against COVID-19, Tunisian PHC missed a crucial opportunity to share its 40 years of experience in the vaccination field, and to benefit from the great number of PHC structures across the country and its proximity to the community in favor of the establishment of parallel and complementary structures that are dedicated to vaccination [24,25]. Despite the pessimistic perception of the experts participating in this study concerning the overall performance of the Tunisian PHC system in the face of COVID-19, they all expressed hope for a better implication in the future. All participants expressed that PHC could face potential upcoming epidemics, provided that major upgrading is conducted, such as the development of the infrastructure of PHC centers, and providing that there are enough human and material resources.

## 5. Conclusions

Even though the role of PHC in the management of epidemics, in general, was not elucidated in both the Alma Ata and Astana declarations, its undeniable role has been documented in previous epidemics. The Tunisian PHC has years of experience with a great number of centers (more than 2000) all across the country, making it accessible to the community. However, the role of the Tunisian PHC system was uncertain. Therefore, a few lessons canbe deduced from the COVID-19 pandemic to manage any potential future ones.

PHC structures and staff should be implicated, starting in the early stages of epidemics. Due to theirgeographical accessibility, screening procedures, early diagnosis, and the capacity of the management of non-severe cases, PHC structures can achieve this.

The national strategy should implicate PHC in the management of epidemics, whether it is by providing them with the technical and medical supplies, or by not omitting their existing services (such as vaccination).

PHC structures can ensure the continuity of essential healthcare services during epidemics. Even though the priorities are shifted during health emergencies, PHC facilities should provide, continuously, the minimum healthcare package.

Researchers should conduct further qualitative studies to explore the limiting factors to better ensure the maintenance of Primary Health Care delivery services.

## Figures and Tables

**Table 1:** Baseline characteristics of participants (N = 25) participants of the study to describe the role of the Tunisian Primary Health Care in the management of the COVID-19 pandemic.

	n (%)
**Profession**	
Medical	23 (92.0)
Administrative	1 (4.0)
Other	1 (4.0)
**Function**	
International	2 (8.0)
National	14 (56.0)
Regional	5 (20.0)
Local	2 (8.0)
Missing	2 (8.0)
**Implication in Primary Health Care**	
Student	5 (20.0)
Practicing	9 (36.0)
Retired	7 (28.0)
Missing	4 (16.0)
**Engagement in COVID-19 fight**	
Engaged	16 (64.0)
Not engaged	8 (32.0)
Missing	1 (4.0)

**Table 2:** Different responses and arguments to the five themes of the study according to the 25 participants of the study whodescribed the role of the Tunisian Primary Health Care in the management of the COVID-19 pandemic.

Questions	Responses	Arguments/Topics Advanced by the Participants
In Tunisia, PHCs have been prepared for the fight against the COVID-19 pandemic with its different waves	PHC was not prepared	The fight against COVID-19 has been concentrated on third line structures; Exhaustion of PHC by previous epidemics; Poor organization of resources; Shortages of protective equipment; Despite the existence of a response and resilience plan for epidemic-prone diseases since 2016, the first line has not benefited from the necessary support; The PHC was deserted by community due to general phobia and staff absenteeism; The organization of the Tunisian health system marginalizes front-line structures.
In Tunisia, PHC has played a significant role in the response to the COVID-19 pandemic and its various waves	PHC played an initial role in the response to the COVID-19 pandemic	**During the first wave** PHC was involved in epidemiological surveillance, information, public awareness, positive diagnosis (clinical examination and samples), and triage of patients. **From the 2nd wave** Appointment of a director general of health that was committed to the front line; Involved when central hospital structures have been overwhelmed Involved as “auxiliaries” to “officials”; Capacity in crisis management and the delivery of first aid; Screening of positive cases and management of non-serious cases; Management of places of isolation/containment for the control of infected cases. Role in information, awareness, and setting up COVID circuits; Important role in anti-COVID vaccination but “ad-hop” mass vaccination centers disrupted the normal functioning of structures, gave a signal of distrust, and constituted a great missed opportunity to improve PHC;
	PHC did not play a role in the response to the COVID-19 pandemic	The response focused on third line health structures (centralization of care); Interruption of essential PHC activity due to the closure of frontline structures, staff absenteeism, and detour to the private sector).
In Tunisia, the national strategy for the fight against COVID-19 in its various waves has marginalized PHC in the dispensation of preparedness and response measures	PHC was not marginalized PHC was marginalized	The response strategy is adapted to WHO recommendations (SSP involved when response possibilities are exceeded by central and hospital structures); Provision of PHC with the necessary material and human resources. **Centralization of the management and support of the pandemic (a matter for specialists)** Difficult access to PHC; Limitation of treatments and remote appointments; Belief that the pandemic is beyond the capacities of PHCs (in terms of human and material resources); The infrastructure does not allow care according to the health protocols in force. **Vaccination** Despite the expertise and recognition by the WHO of the capacities of PHCs in terms of vaccination, there has been the creation of intermediate, regional centers for mass vaccination.
In Tunisia, the national strategy to fight against COVID-19 has weakened the capacities of PHCs in the usual dispensation of the primary care package	COVID-19 has weakened of the delivery of the primary care package	**Political priority regarding COVID-19** A “the other diseases could wait” choice of the Ministry of Health and the presidency of the government; A death by COVID constituted a failure; Focus of all planning and resource management efforts on COVID Reservation of health structures for COVID patients; National strategy based on improvisation and copy/paste; Lack of authority; Reorganization of front-line staff in vaccination centers or in hospital structures (stopgap); Supply of oxygen and treatment for COVID at the expense of antibiotics and drugs for chronic pathologies. **Relaxation of routine activities** Stop consultation and increase in complications; Discontinuation of follow-up of other pathologies such as chronic diseases and cancers; Weakening of immunization and maternal and child health programs. **Social context** General containment; Community phobia; Closure of PHC centers, cessation of any type of non-urgent or routine care, and interruption of outpatient consultations.
In the future, the fight against critical epidemics is a component that can be integrated into the PHC strategy in Tunisia	The fight against critical epidemics should be integrated into the Tunisian PHC strategy	**The management of future epidemics should be a part of PHC functions** PCH is accessible to the community (geography and staff); Community trust in PHC. **Future improvements** Update the preparedness plan for the response and resilience to diseases with high epidemic potential; Strengthen testing and strategic inventory capacities; Infrastructure development; Preparation of personnel.
